# Relationship between clinical outcomes and nerve conduction studies before and after surgery in patients with carpal tunnel syndrome

**DOI:** 10.1186/s12891-021-04771-y

**Published:** 2021-10-16

**Authors:** Masato Ise, Taichi Saito, Yoshimi Katayama, Ryuichi Nakahara, Yasunori Shimamura, Masanori Hamada, Masuo Senda, Toshifumi Ozaki

**Affiliations:** 1 Department of Orthopaedic Surgery, Okayama University Graduate School of Medicine, Dentistry and Pharmaceutical Sciences, 2-5-1, Shikata-cho, Kitaku, Okayama, 700-8558 Japan; 2 Department of Rehabilitation Medicine, Okayama University Hospital, Okayama, Japan; 3 Department of Sports Medicine, Okayama University Graduate School of Medicine, Dentistry and Pharmaceutical Sciences, Okayama, Japan

**Keywords:** Carpal tunnel syndrome, Nerve conduction study, The disability of the arm, shoulder and hand questionnaire, Clinical outcomes

## Abstract

**Background:**

Nerve conduction study (NCS) is the only useful test for objective assessment of carpal tunnel syndrome (CTS). However, the relationship between pre- and postoperative NCS and clinical outcomes was unclear. This study aimed to determine whether pre- and postoperative (6 months) NCS could predict patient-oriented and motor outcomes (6 and 12 months postoperatively) in patients with CTS.

**Method:**

Of the 85 patients with CTS, 107 hands were analyzed from March 2011 to March 2020. All patients underwent open carpal tunnel release and were examined using the disabilities of the arm, shoulder and hand (DASH) questionnaire and grip strength (GS) preoperatively and 6 and 12 months postoperatively. Moreover, NCS was examined preoperatively and 6 months postoperatively. Distal motor latency (DML) and sensory conduction velocity (SCV) were the parameters used for NCS. The correlation coefficient between NCS and DASH or GS was calculated. A receiver operating characteristic curve was utilized to determine the NCS threshold value to predict DASH and GS improvement.

**Results:**

The average scores of GS preoperatively and 6 and 12 months postoperatively were 21.3, 22.3, and 22.8, respectively. On the other hand, the average scores of DASH preoperatively and 6 and 12 months postoperatively were 28.8, 18.3, and 12.2, respectively. The average NCS scores (DML and SCV) preoperatively/6 months postoperatively were 7.3/5.4 and 27.8/36.7, respectively. Preoperative NCS did not correlate with DASH and GS. Postoperative SCV correlated with the change in grip strength (6–12 months, *r* = 0.67; 0–12 months, *r* = 0.60) and DASH (0–12 months, *r* = 0.77). Moreover, postoperative DML correlated with the change in DASH (6–12 months, *r* = − 0.33; 0–12 months, *r* = − 0.59). The prediction for the improvement of GS/DASH achieved a sensitivity of 50.0%/66.7% and a specificity of 100%/100%, at an SCV cutoff score of 38.5/45.0 or above. The prediction for improvement of GS/DASH achieved a sensitivity of 83.3%/66.7% and a specificity of 100%/66.7% at a DML cutoff score of 4.4/4.4 or below.

**Conclusion:**

NCS at 6 months postoperatively can be used to predict the improvement of clinical outcome after 6 months postoperatively in patients with CTS.

**Supplementary Information:**

The online version contains supplementary material available at 10.1186/s12891-021-04771-y.

## Background

Carpal tunnel syndrome (CTS) is the most frequent entrapment neuropathy. In the general population, the prevalence of CTS is reported to be 3.8–10% [1, 2], and among people > 50 years old in Japan, it is reported to be 5.1% [3].

Clinical manifestation and physical findings are used to diagnose CTS. Nerve conduction study (NCS) help to diagnose and decide on the treatment plan (conservative treatment or surgery) as well as predict the surgery prognosis for patients with CTS [4]. Furthermore, NCS is the only useful test for objective CTS assessment. NCS and the clinical symptom severity scale were found to be correlated [5]. Moreover, NCS was reported as a predictor of poor outcomes for surgical release [6]. The clinical success of surgical release for CTS occurs at a rate of 75–90%, whereas the recurrence is reported in 4–57% of cases [7]. Thus, predicting clinical outcomes following surgical release is very useful for patients and surgeons.

However, the relationship between clinical outcomes following surgical release and pre−/postoperative NCS is unclear. In addition, few studies have reported a correlation between NCS and motor outcome (e.g., power or pinch grip). This study aimed to examine the prediction improvement in patient-oriented and motor outcomes after operation using preoperative NCS. Furthermore, whether NCS at 6 months postoperatively can predict clinical outcomes at 12 months postoperatively was investigated.

## Methods

This study analyzed 107 hands of 85 patients with CTS from March 2011 to March 2020. All patients underwent open carpal tunnel release (OCTR), which was performed by two orthopedic hand surgeons. CTS was diagnosed based on the patient’s medical history, thenar muscle atrophy, NCS, and physical examination (e.g., ring-finger splitting, Tinel-like sign, and Phalen’s test). Electrodiagnostic criteria used to diagnose CTS in this study was median distal motor latency from the wrist to abductor pollicis brevis < 4.0 ms [8] and median motor nerve conduction velocity < 45 m/s from the wrist to abductor pollicis brevis and median sensory nerve conduction velocity < 45 m/s in the third digit to wrist [9]. We set the lower limit value of amplitude of compound muscle action potential (CMAP) and sensory nerve action potential (SNAP) of median nerve at wrist stimulation was 3.5 mV and 10 μV respectively [9, 10].

The patients underwent NCS preoperatively and 6 months postoperatively. Grip strength was also measured, and disabilities of the arm, shoulder and hand (DASH) questionnaire was administered before surgery and at the time of outpatient visits at 6 and 12 months postoperatively. Patients with rheumatoid arthritis, artificial dialysis, polyneuropathy, and unavailable NCS waveform at the median nerve were excluded. Finally, 85 hands of 67 patients were included in this study.

This study was approved by the institutional ethics committee at Okayama University Hospital, and informed consent was obtained in the opt-out form on the website.

NCS was conducted using a Neuropack MEB-2300 (Nihon Kohden, Tokyo, Japan). The filter settings were between 20 and 2000 Hz. Stimulation was performed with a square wave pulse of 0.2-ms duration. The compound muscle action potential, distal motor latency, and motor nerve velocity of the median nerve were recorded via orthodromic stimulation at the wrist, 70-mm proximal to recording electrodes placed over the abductor pollicis brevis muscle. Sensory nerve conduction velocity (SCV) was recorded via antidromic stimulation at the wrist, 140-mm proximal to recording electrodes placed over the proximal phalanx of the middle finger using ring electrodes around the proximal and middle phalanxes of the middle finger. The skin temperature of the hand was maintained between 32 °C and 34 °C. Grip strength was measured using Smedley hand dynamometer (Igarashi Ikakougyou, Tokyo, Japan).

Grip strength and DASH were used as parameters for objective and patient-oriented assessments, respectively. DASH is a self-administered questionnaire consisting of a disability/symptom (DASH-DS) scale and two optional modules, namely, work and sport/music modules. Moreover, it was widely used for shoulder, hand, elbow, and wrist problem evaluations. The patients were asked to achieve a score of 1–5 on all 30 items, and the scores increased with the increase in disability. The obtained raw score was then converted to a 0–100 scale. The reliability, validity, and responsiveness of the Japanese version of DASH had equivalent evaluation capacity to those of the DASH original versions [11]. Moreover, this study focused on the score of the DASH-DS scale.

The prediction of functional prognosis following surgical release using NCS was explored using the receiver operating characteristic (ROC) curve. A previous study reported the minimal clinically important differences (MCID) for DASH in patients with isolated tendinitis, arthritis, or nerve compression syndromes [12–14]. Therefore, a cutoff score of > 10 for postoperative clinical improvement in DASH was set. Although no study on the MCID of grip strength in patients with CTS was conducted, the MCID of grip strength was estimated to be 2.69–2.44 kg in the healthy group without conditions [15]. Thus, we set a cutoff value of > 2 kg for postoperative clinical grip strength improvement. Moreover, the ROC curve is a commonly used statistical graphical plot to help evaluate the performance of a diagnostic test by plotting the true-positive (sensitivity) rate against the false-positive (1-specificity) rate at various threshold settings. Youden’s criterion was used to select the ideal cutoff point in order to determine the result of the NCS on the ROC curve. The efficacy of the NCS to predict functional prognosis was described using an area under the curve (AUC), sensitivity, specificity, and positive and negative likelihood ratios.

Statistical analyses were conducted using the Bell Curve (Social Survey Research Information Co., Ltd., Shinjuku-ku, Japan) with the significance level set at < 0.05 for all statistical tests. The correlations between the results of preoperative NCS and clinical outcomes that include grip strength and DASH score were assessed via a nonparametric test (Spearman’s correlation).

## Results

### Participant characteristics

Table 1 presents the patients’ characteristics. This study included 67 (18 men and 49 women) patients, and 85 hands with CTS were analyzed. The average age was 64.5 years ([SD] = 13.3; range = 34–87), and the average symptom duration was 19.5 months (range = 2–72). No reoperation cases were included in this study.

**Table 1:** Clinical characteristics of patients

**Characteristics**	**Values**
Age (years ± SD)	64.5 ± 13.3
Female (n/%)	49/73
Rt operated hand (n)	49
Duration of symptoms (mo, range)	19.5 (2–72)

*mo* months

### NCS and clinical outcomes (grip strength and DASH score)

The mean pre−/postoperative values at 6 and 12 months for grip strength, DASH, and NCS results are presented in Table 2. Grip strength at 6 and 12 months postoperatively improved by an average of 1.9 kg (0–6 months, 95% confidence interval [CI] = 0.5–3.4) and 3.7 kg (6–12 months, 95% CI = 2.6–4.9), respectively, compared with preoperative measurements. Improvement in grip strength between 6 and 12 months postoperatively was higher compared with that between preoperative and 6 months postoperatively. Grip strength in all patients at 12 months postoperatively improved compared with preoperative measurements. The average DASH scores increased to 11.9 (0–6 months, 95% CI = 3.2–20.6) and 14.1 (6–12 months, 95% CI = 2.1–26.1), respectively. The improvement in DASH score between 6 and 12 months postoperatively was also higher compared with that between preoperatively and 6 months postoperatively. The results of NCS were improved between preoperative and 6 months postoperatively.

**Table 2:** Average preoperative and postoperative values

	**Preoperative**		**6 months**		**12 months**	
	**Average (SD)**	**Range**	**Average (SD)**	**Range**	**Average (SD)**	**Range**
Grip	21.3 (10.3)	5–61.8	22.3 (10.5)	8–69.9	22.8 (5.6)	10–35.9
DASH	28.8 (19.5)	0–94.4	18.3 (13.5)	0–49.1	12.2 (8.5)	1–28
Median						
DML	7.3 (6.6)	4–18.2	5.4 (5.9)	0–8.0		
SCV	27.8 (7.3)	9.4–46.8	36.7 (10.7)	0–54.9		
CMAP amplitude	3.8 (3.0)	0.1–12.7	4.2 (3.0)	0–12.0		

*DASH* Disabilities of Arm, Shoulder and Hand, *DML* Distal motor latency (ms), *SCV* Sensory conduction velocity (m/s), *CMAP* Compound muscle action potential (mV), *SNAP* Sensory nerve action potential (μV), *SD* Standard deviation

### Relationships between nerve conduction measures and clinical outcome

Tables 3 and 4 present the correlation coefficients between pre−/postoperative NCS and change in the grip strength and DASH scores at 6 and 12 months postoperatively. A no significant correlation was observed between preoperative NCS (SCV) and change in the grip strength scores (0–6 months, *r* = 0.23; 6–12 months, *r* = 0.21; 0–12 months, *r* = 0.27). Moreover, a significant correlation was not observed between preoperative NCS (SCV) and change in DASH scores (0–6 months, *r* = 0.16; 6–12 months, *r* = − 0.03; 0–12 months, *r* = 0.26). SCV at 6 months postoperatively had modest and significant correlation with the change in grip strength scores (6–12 months, *r* = 0.67; 0–12 months, *r* = 0.60) and DASH score (0–12 months, *r* = − 0.59). NCS (DML) at 6 months postoperatively had no correlation with the change in grip strength scores but modest and significant correlation with the change in DASH scores (6–12 months, *r* = 0.12; 0–12 months, *r* = 0.77).

**Table 3:** Spearman’s correlation coefficients between preoperative nerve conduction measures and difference in grip strength and DASH

**Instrument scale**	**0–6 Difference of GP**	**6–12 Difference of GP**	**0–12 Difference of GP**
DML (ms)	− 0.26	0.07	− 0.15
SCV (m/s)	0.23	0.21	0.27
CMAP amplitude (mV)	0.04	−0.07	−0.36
SNAP amplitude (μV)	0.04	0.14	0.13
**Instrument scale**	**0–6 Difference of DASH score**	**6–12 Difference of DASH score**	**0–12 Difference of DASH score**
DML (ms)	−0.01	− 0.1	− 0.03
SCV (m/s)	0.16	−0.03	0.26
CMAP amplitude (mV)	−0.16	−0.28	− 0.49
SNAP amplitude (μV)	−0.20	0.28	0.11

**Table 4:** Spearman’s correlation coefficients between 6 months postoperative nerve conduction measures and difference in grip strength and DASH

**Instrument scale**	**0–6 Difference of GP**	**6–12 Difference of GP**	**0–12 Difference of GP**
Post 6 month DML	− 0.24	−0.002	− 0.13
Post 6 month SCV	0.31	0.67*	0.60
Post 6 month CMAP	0.41*	0.43	−0.04
Post 6 month SNAP	0.12	0.08	−0.19
**Instrument scale**	**0–6 Difference of DASH score**	**6–12 Difference of DASH score**	**0–12 Difference of DASH score**
Post 6 month DML	0.60*	0.12	0.77*
Post 6 month SCV	−0.23	−0.33	− 0.59
Post 6 month CMAP amplitude	0.26	−0.23	− 0.45
Post 6 month SNAP amplitude	− 0.07	0.09	− 0.38

* *P* < 0.05

Similarly, no significant correlation was observed between amplitudes of preoperative CMAP and SNAP and change in the grip strength and DASH scores at 6 and 12 months postoperatively. The amplitude of CMAP at 6 months postoperatively had modest and significant correlation with the change in grip strength scores (0–6 months, *r* = 0.41; 6–12 months, *r* = 0.43; 0–12 months, *r* = − 0.04).

We also compared the postoperative clinical outcomes between two groups divided by the lower limit of the CMAP amplitude (3.5 mV) at pre- and 6 months post-surgery to investigate the effect of the electrophysiologic pathology on clinical outcomes. There was no significant difference between the two groups (Supplementary Tables 1 and 2).

### Area under the curve in the receiver operating characteristic curve analysis

**Fig. 1:**
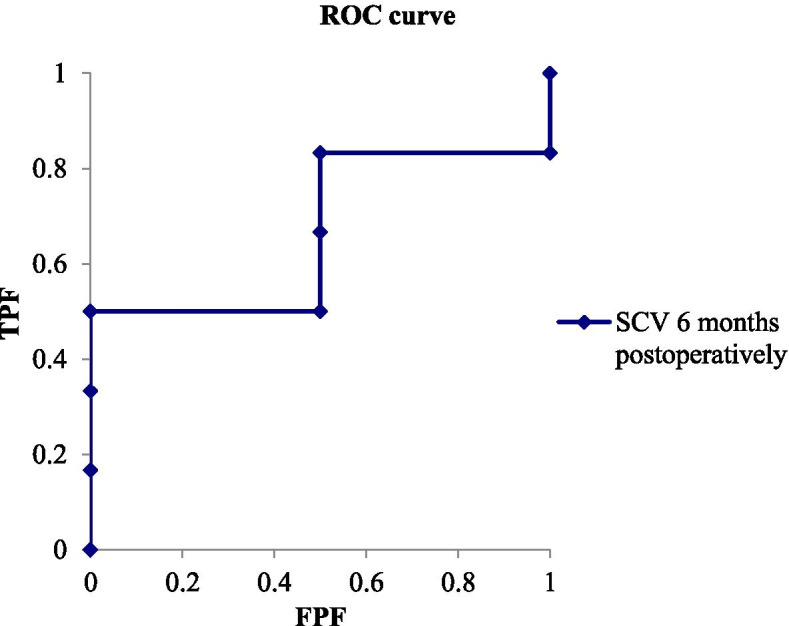
A graph showing the ROC curve for predicting improvement of > 2 kg in grip strength based on SCV at 6 months postoperatively. The actual area under the curve (AUC,0.67 [95% CI, 0.20 to 1.13]). The best cutoff point of SCV for balancing sensitivity and specificity was 38.5

**Fig. 2:**
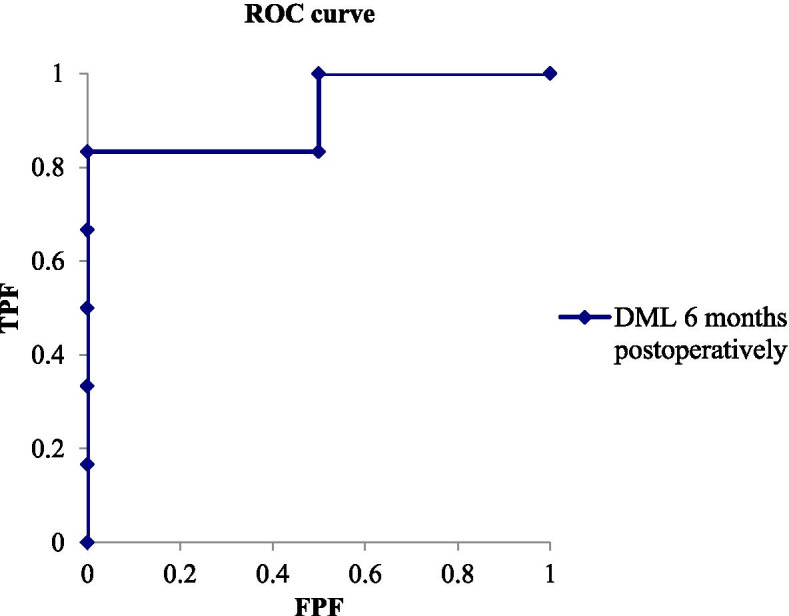
A graph showing the ROC curve for predicting improvement of > 2 kg in grip strength based on DML at 6 months postoperatively. The actual area under the curve (AUC,0.91 [95% CI, 0.69 to 1.15]). The best cutoff point of DML for balancing sensitivity and specificity was 4.4

**Fig. 3:**
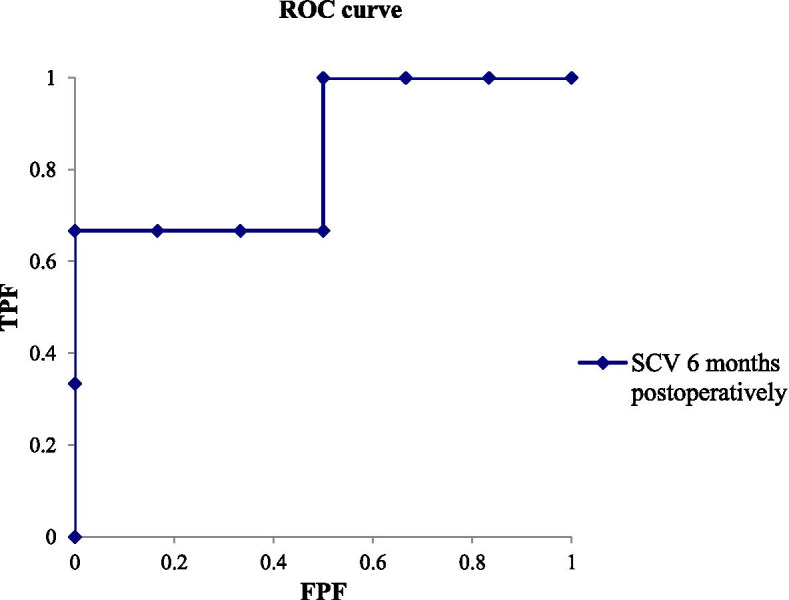
A graph showing the ROC curve for predicting improvement of > 10 in the DASH score based on SCV at 6 months postoperatively. The actual area under the curve (AUC,0.83 [95% CI, 0.48 to 1.19]). The best cutoff point of SCV for balancing sensitivity and specificity was 45.0

**Fig. 4:**
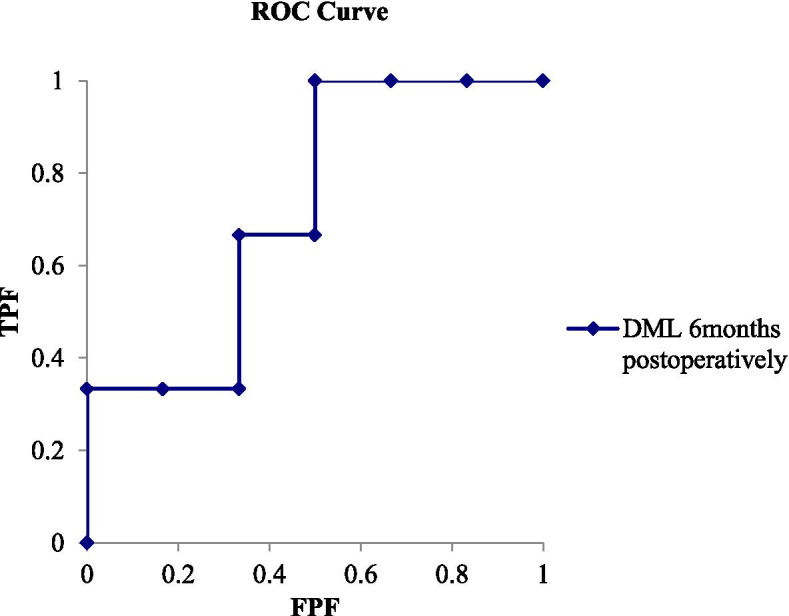
A graph showing the ROC curve for predicting improvement of > 10 in the DASH score based on DML at 6 months postoperatively. The actual area under the curve (AUC,0.72 [95% CI, 0.33 to 1.11]). The best cutoff point of DML for balancing sensitivity and specificity was 4.4

An NCS ROC analysis at 6 months postoperatively (SCV, DML) was conducted to set a cutoff point for predicting grip strength and DASH improvements at 12 months. The SCV results on grip strength demonstrated that the AUC was 0.67 (Fig. 1; 95% CI for AUC, 0.20–1,13). A cutoff value of 38.5 m/s higher for SCV predicts improvement with sensitivity and specificity of 50.0 and 100%, respectively. The DML results indicated that the AUC was 0.91 (Fig. 2; 95% CI for AUC, 0.69–1.15). A cutoff value of 4.4 ms lower for DML predicts improvement with sensitivity and specificity of 83.3 and 100%, respectively. The SCV results on DASH improvement indicated that the AUC was 0.83 (Fig. 3; 95% CI for AUC, 0.48–1,19). A cutoff value of 45.0 m/s lower for SCV predicts improvement with sensitivity and specificity of 66.7 and 100%, respectively. The DML results demonstrated that the AUC was 0.72 (Fig. 4; 95% CI for AUC, 0.33–1,11). A cutoff value of 4.4 ms higher for DML predicts improvement with sensitivity and positive predictive value of 66.7 and 66.7%, respectively.

## Discussion

This retrospective study found a moderate and significant correlation between NCS (SCV) and the change in the grip strength and DASH scores at 6 and 12 months postoperatively. However, a no significant correlation exists between preoperative NCS and clinical outcomes (DASH and grip strength).

The clinical success rate of OCTR was reported to be about 75–90% [16]. Thus, the prediction of postoperative clinical outcomes for patients and surgeon is beneficial. For surgeons, the prediction of the postoperative clinical course is helpful for the selection of patients with poor prognoses as well as explanations for patients and considerations for operation. Several studies were conducted using NCS and patient-reported outcomes (PRO) to predict prognosis following operation [6, 17, 18]. However, there are conflicting reports on the severity of conduction disturbances and surgery outcomes. Heybeli et al. reported that NCS improvements did not correlate with improvement in the self-administered Boston Questionnaire preoperatively and 6 months preoperatively [19]. Itubo et al. also reported that no significant correlation was observed between the preoperative and 3 months postoperative patient-oriented questionnaires and preoperative and 3 months postoperative NCS [18]. On the other hand, Bland et al. reported that preoperative NCS correlated with postoperative clinical outcomes and that patients with middle-grade abnormalities achieved better results compared with those with severe abnormalities [6]. This study demonstrated a no significant correlation between preoperative NCS and improvement in clinical outcomes (grip strength and DASH). In addition, the follow-up period was short (within 6 months) in most studies that investigated the correlation between postoperative clinical outcomes and NCS [13, 18, 20]. Furthermore, this study revealed that a significant and modest correlation was observed between NCS (SCV) at 6 months postoperatively and the change in grip strength and DASH at 12 months postoperatively.

Setting a threshold value is useful for determining CTS severity and predicting clinical outcome prognosis following operation. Concerning the threshold value of NCS, Bland et al. reported that distal motor latency to abductor pollicis brevis < 6.5 ms was utilized to evaluate the severity of CTS [21]. In addition, Lo et al. reported that a cutoff value of 6.0 ms or lower for preoperative sensory latency predicts a good outcome (in terms of paresthesia score) with sensitivity/certainty and positive predictive values of 84.6 and 86.8%, respectively, in patients with CTS following endoscopic carpal tunnel syndrome surgery [13]. This study revealed the improvement in grip strength/DASH at 12 months postoperatively in patients with > 38.5 m/s (SCV) and < 4.4 ms (DML)/ > 45.0 m/s (SCV) and < 4.4 ms (DML) at 6 months postoperatively, respectively. To the best of our knowledge, this study is the first to reveal the NCS threshold value to predict PRO and grip strength improvement following surgery. Nolan et al. reported that even at 66 months postoperatively, only 50% of hands demonstrated normal DML [22]. This study demonstrated that 2.4%/1.2% of hands exhibited normal DML/SCV preoperatively, and 23%/44% of hands exhibited normal DML/SCV at 6 months postoperatively. Even if the results of NCS at 6 months postoperatively are not normal, the results of this study can help predict improvements in grip strength and DASH. Thus, these results suggested that NCS at 6 months postoperatively can be used not only to evaluate the degree of postoperative NCS improvement but also to predict the improvement in grip strength/DASH at 12 months postoperatively.

This study used grip strength for postoperative clinical motor evaluation. However, there is no consensus as to what method for motor function assessment of patients with CTS following surgical treatment is the most useful [23]. However, grip strength was one of the major objective motor outcomes. Yoshida et al. reported the grip strength-related disability of the upper extremity in patients with CTS [24]. Some other studies also used grip strength as motor outcomes in patients with CTS following surgery [25–29].

Grip strength at 6 months postoperatively decreased in 40% of the patients compared with the preoperative measurement. However, grip strength improved from preoperative levels in all patients who were followed up at 12 months postoperatively. Scar pain or tenderness may influence grip strength weakness at 6 months postoperatively.

The follow-up period in this study is limited to 1 year. Thus, whether NCS at 6 months postoperatively will be related to long-term (more than 1 year) follow-up was not determined. According to the preceding study, the clinical outcomes following surgical release improved up to 6 months postoperatively. The same outcome was obtained after 6 years [30]. De Kleermaeker et al. investigated the long-term outcome after operation in patients with CTS at 8 months postoperatively and the median follow-up at 9 years. They reported that the most important factor associated with the long-term outcome is treatment outcome after about 8 months, and its effect persists after 9 years [16]. Atroshi et al. also reported the clinical outcome at 1 year postoperatively, which was maintained at the final follow-up (11–16 years) [31]. Although the evaluation of outcomes and NCS in this study was conducted within a year, clinical outcome prognosis at 1 year postoperatively may represent a long period of functional prognosis of more than 1 year.

Neurophysiological severity of CTS median nerve may affect postoperative clinical outcomes. The postoperative CMAP amplitude had significant correlation with change in the grip strength at 6 months postoperatively. However, there was no significant difference in postoperative clinical outcomes between the two groups divided by the lower limit of CMAP amplitudes in this study. We evaluated only the amplitude, though the amplitude and duration of CMAP are required to correctly distinguish between axonal loss and demyelinating conduction block. The limitation may have influenced on the outcomes.

Patients with unavailable NCS (MCV and SCV) waveform at the median nerve were excluded. Therefore, the very severe cases with no available waveform were not included in this study. Thus, the result of this study may not be applied to patients with very severe CTS.

## Conclusions

This study found that preoperative NCS is not used to predict postoperative clinical outcomes. However, the results of the 6 months postoperative NCS can predict the improvement in the DASH score at 12 months postoperatively.

## Supplementary Information

**Additional file 1: Supplementary Table 1.** Comparison of average difference of grip strength and DASH between two groups divided by the lower limit of preoperative CMAP amplitudes. **Supplementary Table 2.** Comparison of average difference of grip strength and DASH between two groups divided by the lower limit of 6 months postoperative CMAP amplitudes.

## Data Availability

The datasets used and analyzed during the current study are available from the corresponding author on reasonable request.
