# Supplementary material for: Relationship between clinical outcomes and nerve conduction studies before and after surgery in patients with carpal tunnel syndrome
Source: BMC Musculoskelet Disord. 2021 Oct 16;22:882. doi: 10.1186/s12891-021-04771-y (PMC8520296; doi:10.1186/s12891-021-04771-y)
Supplement: Supplementary file 1 — Additional file 1: Supplementary Table 1. Comparison of average difference of grip strength and DASH between two groups divided by the lower limit of preoperative CMAP amplitudes. Supplementary Table 2. Comparison of average difference of grip strength and DASH between two groups divided by the lower limit of 6 months postoperative CMAP amplitudes. [file 12891_2021_4771_MOESM1_ESM.pptx]

## Slide 1
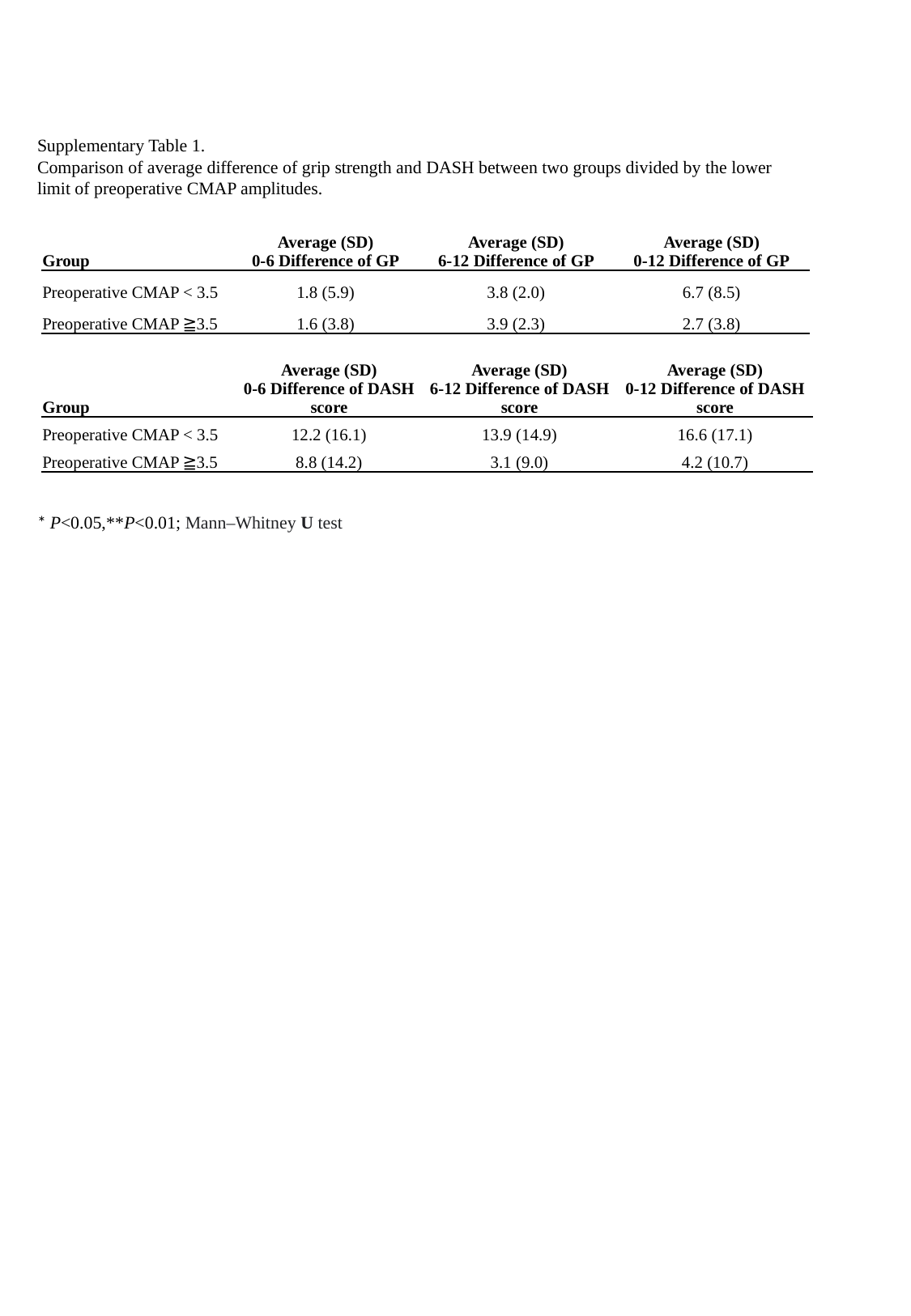

Supplementary Table 1.
Comparison of average difference of grip strength and DASH between two groups divided by the lower limit of preoperative CMAP amplitudes.
| Group | Average (SD) 0-6 Difference of GP | Average (SD) 6-12 Difference of GP | Average (SD) 0-12 Difference of GP |
| --- | --- | --- | --- |
| Preoperative CMAP < 3.5 | 1.8 (5.9) | 3.8 (2.0) | 6.7 (8.5) |
| Preoperative CMAP ≧3.5 | 1.6 (3.8) | 3.9 (2.3) | 2.7 (3.8) |
| Group | Average (SD) 0-6 Difference of DASH score | Average (SD) 6-12 Difference of DASH score | Average (SD) 0-12 Difference of DASH score |
| --- | --- | --- | --- |
| Preoperative CMAP < 3.5 | 12.2 (16.1) | 13.9 (14.9) | 16.6 (17.1) |
| Preoperative CMAP ≧3.5 | 8.8 (14.2) | 3.1 (9.0) | 4.2 (10.7) |
* P<0.05,**P<0.01; Mann–Whitney U test

## Slide 2
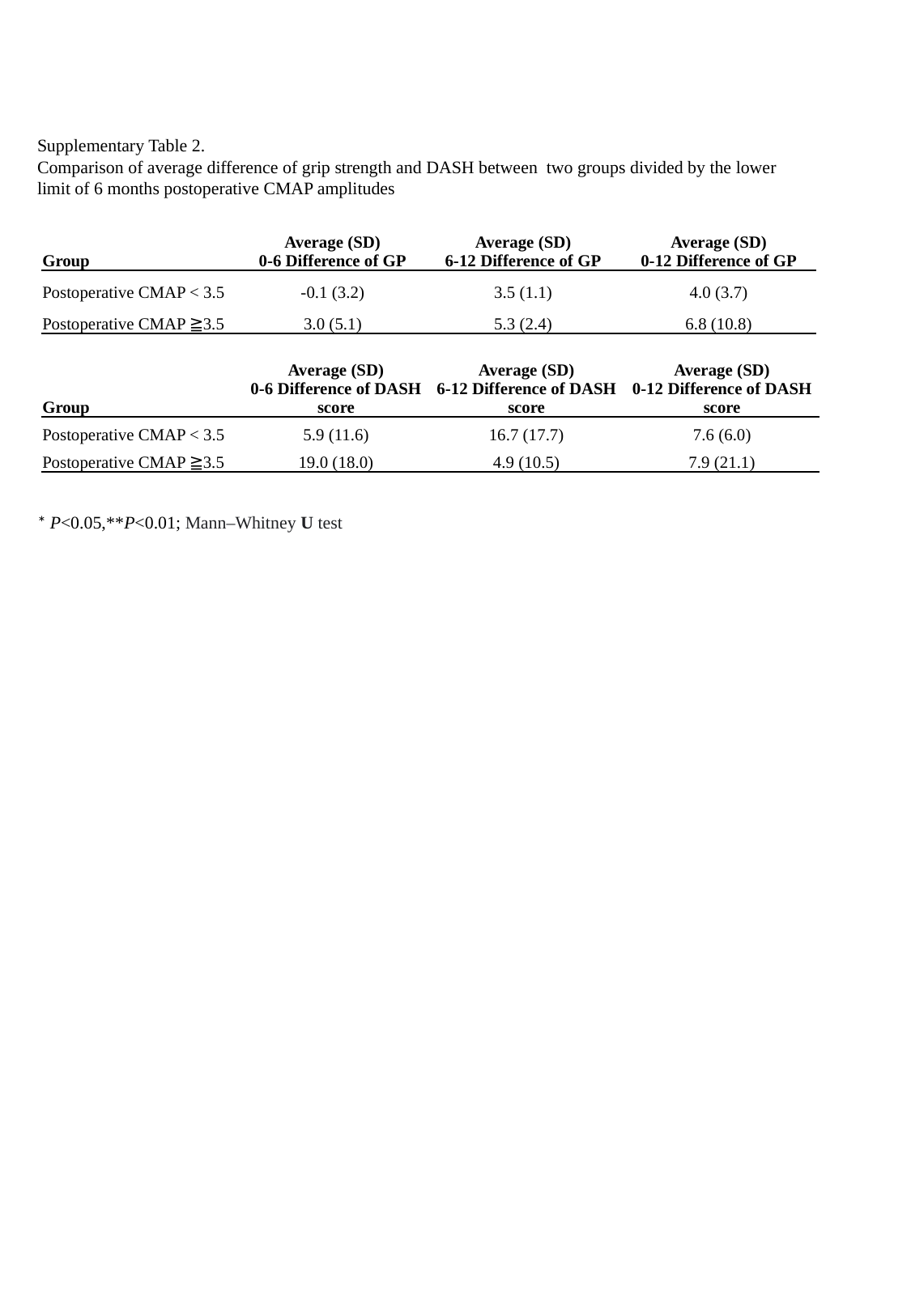

Supplementary Table 2.
Comparison of average difference of grip strength and DASH between two groups divided by the lower limit of 6 months postoperative CMAP amplitudes
| Group | Average (SD) 0-6 Difference of GP | Average (SD) 6-12 Difference of GP | Average (SD) 0-12 Difference of GP |
| --- | --- | --- | --- |
| Postoperative CMAP < 3.5 | -0.1 (3.2) | 3.5 (1.1) | 4.0 (3.7) |
| Postoperative CMAP ≧3.5 | 3.0 (5.1) | 5.3 (2.4) | 6.8 (10.8) |
| Group | Average (SD) 0-6 Difference of DASH score | Average (SD) 6-12 Difference of DASH score | Average (SD) 0-12 Difference of DASH score |
| --- | --- | --- | --- |
| Postoperative CMAP < 3.5 | 5.9 (11.6) | 16.7 (17.7) | 7.6 (6.0) |
| Postoperative CMAP ≧3.5 | 19.0 (18.0) | 4.9 (10.5) | 7.9 (21.1) |
* P<0.05,**P<0.01; Mann–Whitney U test
